# Depression and Interpersonal Problems in Adolescents: Their Relationship With Alexithymia and Coping Styles

**Published:** 2014

**Authors:** Masoud Talebi Joybari

**Affiliations:** 1 Department of Psychology, School of Education Science & Psychology, Mohaghegh Ardabili University, Ardabil, Iran.

**Keywords:** Adolescents, Alexithymia, Coping Styles, Depression, Interpersonal Problems

## Abstract

**Objective:**

The aim of the present research was to determine whether depression and interpersonal problems had relationships with alexithymia and coping styles in adolescents.

**Methods:**

The study population was randomly selected from all of the adolescent students in the schools of Sari in Iran; 441 adolescents (228 boys and 213 girls) were included in the study. The participants completed the Toronto Alexithymia Scale, the Coping Inventory for Stressful Situations, the Inventory of Interpersonal Problems, and the Beck Depression Inventory. The data was analyzed using descriptive and inferential statistics and was expressed in means, standard deviations, and Pearson correlation coefficient.

**Results:**

Alexithymia was related to depression and interpersonal problems; the adolescents who defined themselves as more alexithymic obtained higher scores in depression and interpersonal problems than the adolescents who classified themselves as less- and non-alexithymic. Furthermore, coping styles were related to depression and interpersonal problems. Regression analyses showed that both alexithymia and coping styles accounted for a unique and significant proportion of the variance in depression and interpersonal problems in adolescents.

**Conclusion:**

These findings support the positive correlation of alexithymia and maladaptive coping styles with depression and interpersonal problems.

## Introduction

Adolescence has been identified as a particularly demanding period of life with a variety of life challenges or developmental tasks (1, 2). The successful resolution of each task results in a healthier psychological system better prepared to meet the demands of the next developmental challenge. In contrast, the inadequate resolution of such tasks facilitates the integration of maladaptive strategies that render the individual less capable of resolving future developmental issues. Thus, early competence fosters later competence; conversely, early incompetence begets later incompetence (3). Depression can be seen as an unsuccessful response to such developmental challenges. Therefore, the development of adaptive coping strategies during this period is believed to lay the foundation for successful adjustment in adulthood (4). Interpersonal problems refer to difficulties that individuals have in relating to others and that cause or are related to significant distress (5).

Preliminary evidences support the relationship between alexithymia and maladapted patterns of coping and depression and higher non-satisfaction with life, maladaptive behaviors, and interpersonal problems (6-15).

Some individuals are incapable of identifying their emotions. Sifneos was the first to label this problem ‘‘alexithymia” (16). Alexithymia is a multidimensional personality construct characterized by three core features: a) difficulty identifying emotions and distinguishing between emotions and bodily sensations; b) difficulty describing or communicating emotions to others; and c) an externally oriented style of thinking (17, 18). Multiple etiologies, from biological to psychological, have been proposed for the development of alexithymia (19, 20). Recent researches support a social learning model of alexithymia in which parents who have difficulties regulating their own emotions (i.e., high levels of alexithymia) also have more difficulty attending to and interpreting their children’s emotions, thus not being able to teach their children how to regulate their own emotions (21-23). Amongst adults, empirical evidence suggests that alexithymia is related to a wide range of psychological problems including poor emotion regulation strategies and higher rates of somatic illness and disease (23).

Coping strategies play a major role in an individual’s physical and mental health when confronted with stressful situations. The construct of coping has been defined as the cognitive and behavioral efforts of an individual to manage the external and/or internal demands encountered during specific stressful situation (24). Coping responses have been categorized into three broad higher-order functions including problem-focused (task-oriented) coping, emotion-focused coping, and avoidance-focused coping (25). Problem-focused coping refers to actions that are employed in order to reduce demands or increase skills to manage demands. Emotion-focused coping represents the actions that are employed in order to change the meaning of a stressful situation as well as to regulate the resulting negative emotions. Finally, avoidance-focused coping refers to actions that are employed in order to disengage oneself from a stressful situation.

The few studies exploring alexithymia in younger age groups have used similar measures as those for adults and have shown it to be linked to poor socio-emotional and physical outcomes such as obesity, the experience of posttraumatic responses following medical treatments, and greater dissociative tendencies (26-28).

In a study conducted on American adolescents, researchers found that problem-oriented and logical coping styles could be indicators of a reasonably good health, and avoidant coping styles and emotional inefficiencies were associated with the imperfect indicator of disease. Moreover, men are mentally less competent than women and it can be concluded that mental health of adolescent girls and their problem-focused and logical coping styles could have a positive effect on their mental health (29). Another study showed that coping strategies negatively associated with aberrant behavior disorders and diseases harmful to personal health, but avoidant coping strategy was positively associated with a variety of psychological and personality problems (30).

A study by Zarei and Besharat showed that alexithymia had a significant positive association with interpersonal problems. Results of regression analysis revealed that alexithymia and its components can predict students' interpersonal problems regarding assertiveness, sociability, submissiveness, intimacy, responsibility, and controlling. It was concluded that alexithymia is associated with interpersonal problems (31).

A study on high school students in Canada has shown that alexithymia is the best predictor of behavioral problems and social anxiety; the excitement management education driven by reduced interpersonal behavioral problems can regulate difficulties in identifying and describing feelings and emotions which in turn help an individual communicate effectively with those around him/her (10).

A recent longitudinal study demonstrated that alexithymia is a predictor of poor socio-emotional outcomes 1 year later (32). In contrast, adolescents with low levels of alexithymia tend to display lower rates of anxiety and depression and report higher satisfaction with life (33). Quite surprisingly, no studies have examined whether and to what extent adolescents are able to distinguish alexithymia from other self-evaluative measures. The purpose of the present study was to determine whether adolescents who classify themselves as high-alexithymic report higher levels of depressive and interpersonal problems than adolescents who classify themselves as non-alexithymic, whether emotion-oriented coping skills are accompanied by higher levels of depression and interpersonal problems, and whether high-alexithymic personality and emotion-oriented coping skills both account for a unique proportion in the development of depression and interpersonal problems.

## Materials and Methods

In a cross-sectional study, the subjects were asked to complete a self-rating questionnaire. The study samples were randomly selected from all of the adolescent students in the schools of Sari in Iran. The population of the study consisted of 441 adolescents (228 boys and 213 girls).

***Questionnaires***

*The Toronto Alexithymia Scale-20:* The Toronto Alexithymia Scale is a 20 item self-report measure. Each item is rated on a 5-point Likert scale ranging from 1 (strongly disagree) to 5 (strongly agree). It provides a total alexithymia score and also ratings of the 3 sub-scales of difficulty identifying feelings, difficulty describing feelings, and externally oriented thinking. The validity and reliability coefficients of the original questionnaire are 0.79 for total alexithymia, 0.81 for difficulty identifying feelings, 0.78 for difficulty describing feelings, and 0.76 for externally oriented thinking (20). The TAS-20 has demonstrated good psychometric properties which were reported by assessing a sample of 587 Iranian undergraduate students. The Persian version of this questionnaire has been evaluated and reliability coefficients for total alexithymia, and the 3 subscales of difficulty identifying feelings, difficulty describing feelings, and externally oriented thinking were, respectively, 0.85, 0.79, 0.68, and 0.74 indicating good internal consistency of the scale (34).

*Coping Inventory for Stressful Situations (CISS):* This questionnaire was designed by Endler and Parker (1990) and consists of 48 questions dividing coping behavior into 3 main areas. Subjects respond to the questions on a 5-point Likert scale ranging from 1 (not at all) to 5 (very much). The CISS is made up of 3 sub-scales; 16 questions for task-oriented coping behavior, 16 questions for emotion-oriented coping behavior, and 16 questions for avoidance-oriented coping behavior. 

Adequate psychometric properties of the English and Persian versions of the scale have been evaluated. Validity and reliability coefficients of the original questionnaire are 0.66; reliability coefficient has been reported to be 0.83 for the style-oriented coping, 0.80 for emotion-oriented coping, and 0.72 for avoidance-oriented coping (23). Validity and reliability coefficients of the Persian version of the questionnaire are 0.67, and reliability coefficient of style-oriented, emotion-focused, and avoidance-oriented copings are 0.83, 0.80, and 0.72, respectively (35).

*Beck Depression Inventory (BDI):* Depression was measured using the Turkish version of the Beck Depression Inventory, which consists of 21 items. The adolescents were asked to choose 1 sentence, from a group of 4, that best described their feelings within the last 2 weeks (e.g., “I do not feel like a failure, I have failed more than I should have, As I look back I see a lot of failures, I feel I am a total failure as a person). The responses were summed across 20 items (one item on suicide was deleted) so that higher scores indicated a higher incidence of depressive symptoms. In the present sample, the females and the males significantly differed in depression scores [t (268) = 2.64; p = 0.009]. Contrary to some findings of researches on depression in the Turkish culture, the females (mean (±SD) = 13.69 (± 8.00)) were found to be more depressed than the males (11.04 (± 8.31)). Validity and reliability coefficients of the original questionnaire were 0.74 (36). Validity and reliability coefficients of the Persian version of the questionnaire were 0.66 (37).

*Inventory of Interpersonal Problems (IIP-60):* This is an abbreviated version of the Inventory of Interpersonal Problems (IIP-127; Horowitz et al., 1988) that consisted of 60 items chosen on the basis of an initial factor analysis of the IIP-127. The inventory is divided into 2 sections; the first section is composed of items starting with the phrase “It is hard for me to”, and the second section of items starting with the phrase “The following are things you do too much”. Items are rated on a 5-point Likert scale, from 0 (not at all) to 4 (extremely) in response to the question “How much have you been distressed by this problem?”. The IIP has been shown to have good test-retest reliability, high internal consistency, and sensitivity to changes during brief psychotherapy. Validity and reliability coefficients of the original questionnaire were 0.87 (15). Validity and reliability coefficients of the Persian version of the questionnaire were 0.81 (38).

***Data collection***

The questionnaires were distributed in the schools and the participants completed the set of questionnaires anonymously in their classrooms. The purpose of the study and the method of filling the questionnaires were explained to each of the subjects. The participants were ensured of the confidentiality of the responses. The mean time taken for completing the questionnaires was 35 minutes. The participation rate was very high (i.e., > 95%). The mean age of the adolescents was 14.8 (± 0.8) years= (12–16 years). In this study, SPSS for Windows (version 16; SPSS Inc., Chicago, IL, USA) was used for data analysis. The differences in the variables between the two groups were evaluated using multivariate analysis of variance (MANOVA) test. In addition, the Pearson correlation test was used in order to examine the relationship between the variables.

## Results

One-way ANOVA was used to examine differences between the high-alexithymic adolescents, the low-alexithymic adolescents, and the non-alexithymic adolescents in terms of coping strategies, depression, and interpersonal problems. As shown in table 1, significant differences in coping strategies were found between the 3 subject groups. Post-hoc analysis of the significant differences found in the task-oriented coping showed that the non-alexithymic group scored significantly higher than the low- and the high-alexithymic groups (p < 0.001). The low- and the high-alexithymic groups did not differ from one another. With regard to the emotion-oriented coping, the high-alexithymic adolescents scored higher than the low- and the non-alexithymic groups (p < 0.001). Again, the low- and the non-alexithymic groups did not differ from one another. In the avoidance-oriented coping, the high- and the low-alexithymic adolescents scored higher than the non-alexithymic adolescents (p < 0.001), but they did not differ from one another. About depression and interpersonal problems, the low- and the high-alexithymic adolescents scored higher than the non-alexithymic adolescents (p < 0.001); the low- and the high-alexithymic groups did not differ from one another.

Partial correlations (corrected for gender) between alexithymia coping styles, depression, and interpersonal problems are displayed in table 2. As shown, depression and interpersonal problem scores were negatively correlated with the non-alexithymic group and task-oriented coping. Furthermore, positive and mostly significant relationships were found between depression and interpersonal problems scores and scores of low-alexithymic, high-alexithymic, and both emotion-oriented coping and avoidance-oriented coping.

**Table 1:** Mean task-oriented coping, emotion oriented coping, and avoidance-oriented coping scores of the adolescents who classified themselves as high–alexithymic, low-alexithymic, and non-alexithymic

	**Attachment style**					
	**High-alexithymic** **(n = 84)**	**Low-alexithymic** **(n = 146)**	**Non-alexithymic** **(n = 191)**	**F**	**P**	**Post-hoc Scheffe test**
	**Mean (± SD)**	**Mean (±SD)**	**Mean (±SD)**			
**Task-oriented coping**	048.21 (± 7.41)	049.74 (± 08.36)	058.36 (± 9.360)	20.41	0.001	Non > Low & High
**Emotion-oriented coping**	051.28 (± 8.21)	045.65 (± 07.64)	043.21 (± 6.54)	12.21	0.001	High > Low & Non
**Avoidance-oriented coping**	054.74 (± 9.59)	051.94 (± 10.32)	045.65 (± 8.79)	14.65	0.001	High & Low > Non
**Depression**	041.36 (± 6.35)	028.39 (± 08.41)	017.47 (± 7.64)	18.36	0.001	Non < Low < High
**Interpersonal problems**	155.36 (± 12.36)	120.47 (± 13.65)	116.28 (± 12.84)	19.71	0.001	High > Low & Non

**Table 2:** Partial correlations (corrected for gender) between alexithymia, coping styles, and depression and interpersonal problems scores (n = 441)

	**Depression**	**Interpersonal problems**
**Non-alexithymic**	-0.48	-0.056
**Low-alexithymic**	00.39	00.43
**High-alexithymic **	00.57	00.64
**Task-oriented coping**	-0.59	-0.67
**Emotion oriented coping**	00.48	00.57
**Avoidance-oriented coping**	00.42	00.64

## Discussion

The present study examined the relationships between alexithymia and coping styles and depression and interpersonal problems as psychological disorders in a sample of adolescent students. Several studies have demonstrated relationships between the personality construct of alexithymia and various psychological disorders (21, 39-42). In general, our findings are consistent with previous researches, indicating a positive relationship between alexithymia and depression in a variety of clinical and nonclinical populations (43-45). Within the general population, alexithymia has also been associated with a variety of interpersonal problems, including social isolation, insecure attachment, and maladaptive behaviors (9, 12, 22, 27, 46).

The present study found that alexithymia, as measured by TAS-20 in adolescent samples, is positively associated with the use of emotion- and avoidance-oriented coping strategies and negatively associated with the use of task-oriented coping strategies. The high-alexithymic students scored significantly higher on the emotion- and avoidance-oriented coping and significantly lower on the task-oriented coping than the low- and the non-alexithymic students. These findings are consistent with those of the previous studies in which alxithymia was found to be associated with maladaptive coping styles (6). 

There is empirical evidence that alexithymia is associated with problems in processing emotional information. Moreover, a lack of empathy has been observed in alexithymic people. Their inability to empathize influences their interpersonal relationships, especially sociability and intimacy. Thus, it can be concluded that alexithymia increases interpersonal problems by causing deficiency in empathy and sympathy. Alexithymia is the equivalent of having problems in emotional self–regulation; when emotional information cannot be perceived and evaluation of cognitive processing is impaired, the person becomes helpless and deranged emotionally and cognitively. This dysfunction causes many problems in social relations for the individual. On the basis of these two explanations, interpersonal problems may be induced directly by dysfunction in cognitive processing of emotional information and regulation of emotions, or by dysfunction in individual mental health. Another explanation is the structural explanation; the structures of triad factors of alexithymia, which have integration and continuity (the characteristic that internal validity of items of alexithymia scale confirms) reinforce and complete other roles by inter influence.

As a consequence, individuals scoring high on measures of alexithymia are unable to think about problems of stressful situations, to analyze those problems, and to find appropriate solutions for the problems. Examples of task-oriented coping strategies assessed by the CISS include these cognitive characteristics and may explain the negative association between alexithymia and this kind of coping behaviors. It is also suggested that high-alexithymic individuals have a limited ability to think about and use emotions to cope effectively with stressful situations (6). When high-alexithymic individuals are not able to use cognitive strategies to modulate their emotional states evoked by stressful situations, the possibility of using maladaptive coping behaviors will increase. The present study also found that alexithymia was positively associated with interpersonal problems. The high-alexithymic students experienced more interpersonal problems than low- and non-alexithymic students. This is consistent with the previous findings that showed alexithymic individuals show higher levels of interpersonal problems.

In short, this study showed that alexithymia and coping styles were positively correlated with depressive and interpersonal problems in adolescents. These results can be useful in interventional treatments based on processing of emotional information and regulation of emotions. At a theoretical level, these findings can confirm current theories on alexithymia and coping styles in depression and interpersonal problems, and pose new questions on this topic.

Assessment of depressive, interpersonal problems, alexithymia, and coping styles based on self-reports of subjects may show the probable biases. Conducting a similar study in other groups may help to evaluate the effect of emotion regulation on emotional disorders, depression, coping styles, and interpersonal problems.

## Notes

**Declaration of interest:** None.
